# Supplementary material for: Molecular surveillance of insecticide resistance in Phlebotomus argentipes targeted by indoor residual spraying for visceral leishmaniasis elimination in India
Source: PLoS Negl Trop Dis. 2023 Nov 8;17(11):e0011734. doi: 10.1371/journal.pntd.0011734 (PMC10659200; doi:10.1371/journal.pntd.0011734)
Supplement: S1 Table — (DOCX) [file pntd.0011734.s001.docx]

**S1 Table. Generalised linear model results for allele and genotype frequencies as predicted by district and year.**

Odds ratios significantly higher than the reference are shown in red and those significantly lower in blue type.

|  | **L_allele_freq** | | | | **S_allele_freq** | | | | **F_allele_freq** | | | |
| --- | --- | --- | --- | --- | --- | --- | --- | --- | --- | --- | --- | --- |
| source | odds ratio | 95% lower C.I. | 95% upper C.I. | P-value | odds ratio | 95% lower C.I. | 95%  upper C.I. | P-value | odds ratio | 95% lower C.I. | 95%  upper C.I. | P-value |
| intercept | 0.13 | 0.11 | 0.15 |  | 0.91 | 0.84 | 0.99 |  | 0.55 | 0.50 | 0.60 |  |
| *District* |  |  |  |  |  |  |  |  |  |  |  |  |
| Darjeeling | 7.78 | 6.68 | 9.07 | <0.001 | 0.10 | 0.09 | 0.12 | <0.001 | 1.33 | 1.19 | 1.49 | <0.001 |
| East Champaran | 11.60 | 10.13 | 13.28 | <0.001 | 0.59 | 0.54 | 0.64 | <0.001 | 0.15 | 0.13 | 0.17 | <0.001 |
| Godda | 5.25 | 4.58 | 6.01 | <0.001 | 0.31 | 0.28 | 0.34 | <0.001 | 1.09 | 1.00 | 1.19 | 0.045 |
| Gopalganj | 1.57 | 1.34 | 1.84 | <0.001 | 1.90 | 1.72 | 2.10 | <0.001 | 0.39 | 0.35 | 0.44 | <0.001 |
| Katihar | 6.59 | 5.69 | 7.62 | <0.001 | 0.22 | 0.20 | 0.25 | <0.001 | 1.09 | 0.99 | 1.21 | 0.093 |
| Muzaffarpur | 2.28 | 1.97 | 2.63 | <0.001 | 1.33 | 1.22 | 1.46 | <0.001 | 0.49 | 0.45 | 0.54 | <0.001 |
| Purnia | 4.16 | 3.58 | 4.82 | <0.001 | 0.42 | 0.38 | 0.47 | <0.001 | 1.04 | 0.94 | 1.16 | 0.435 |
| Samastipur (reference) | |  |  |  |  |  |  |  |  |  |  |  |
| *Year* |  |  |  |  |  |  |  |  |  |  |  |  |
| 2017 (reference) |  |  |  |  |  |  |  |  |  |  |  |  |
| 2018 | 0.65 | 0.60 | 0.70 | <0.001 | 1.14 | 1.06 | 1.22 | <0.001 | 1.35 | 1.25 | 1.46 | <0.001 |
| 2019 | 0.68 | 0.64 | 0.73 | <0.001 | 1.13 | 1.05 | 1.21 | <0.001 | 1.32 | 1.23 | 1.42 | <0.001 |
| 2020 | 0.67 | 0.61 | 0.73 | <0.001 | 1.27 | 1.17 | 1.38 | <0.001 | 1.18 | 1.08 | 1.30 | <0.001 |
| 2021 | 1.10 | 1.01 | 1.19 | 0.027 | 0.87 | 0.80 | 0.95 | 0.002 | 1.01 | 0.93 | 1.11 | 0.767 |

|  | **LL_freq** | | | | **LS_freq** | | | | **LF_freq** | | | |
| --- | --- | --- | --- | --- | --- | --- | --- | --- | --- | --- | --- | --- |
| source | odds ratio | 95%  lower C.I. | 95%  upper C.I. | P-value | odds ratio | 95% lower C.I. | 95%  upper C.I. | P-value | odds ratio | 95% lower C.I. | 95%  upper C.I. | P-value |
| intercept | 0.00 | 0.00 | 0.01 | <0.001 | 0.01 | 0.01 | 0.02 | <0.001 | 0.29 | 0.25 | 0.34 | <0.001 |
| *District* |  |  |  |  |  |  |  |  |  |  |  |  |
| Darjeeling | 3.58 | 1.16 | 11.02 | 0.026 | 1.15 | 0.52 | 2.51 | 0.73 | 26.44 | 21.47 | 32.55 | <0.001 |
| East Champaran | 154.71 | 57.81 | 414.03 | <0.001 | 59.48 | 35.57 | 99.44 | <0.001 | 0.42 | 0.35 | 0.51 | <0.001 |
| Godda | 16.23 | 6.02 | 43.73 | <0.001 | 6.10 | 3.60 | 10.35 | <0.001 | 6.63 | 5.68 | 7.73 | <0.001 |
| Gopalganj | 11.23 | 4.04 | 31.21 | <0.001 | 17.10 | 10.10 | 28.95 | <0.001 | 0.33 | 0.26 | 0.42 | <0.001 |
| Katihar | 21.16 | 7.74 | 57.83 | <0.001 | 7.55 | 4.37 | 13.02 | <0.001 | 8.13 | 6.83 | 9.69 | <0.001 |
| Muzaffarpur | 23.63 | 8.76 | 63.73 | <0.001 | 9.53 | 5.62 | 16.15 | <0.001 | 0.87 | 0.73 | 1.04 | 0.130 |
| Purnia | 12.00 | 4.32 | 33.32 | <0.001 | 7.69 | 4.46 | 13.25 | <0.001 | 4.17 | 3.51 | 4.95 | <0.001 |
| Samastipur (reference) | |  |  |  |  |  |  |  |  |  |  |  |
| *Year* |  |  |  |  |  |  |  |  |  |  |  |  |
| 2017 (reference) |  |  |  |  |  |  |  |  |  |  |  |  |
| 2018 | 0.71 | 0.60 | 0.85 | <0.001 | 0.67 | 0.58 | 0.77 | <0.001 | 0.59 | 0.53 | 0.67 | <0.001 |
| 2019 | 0.77 | 0.66 | 0.90 | 0.001 | 0.73 | 0.64 | 0.83 | <0.001 | 0.58 | 0.53 | 0.65 | <0.001 |
| 2020 | 0.80 | 0.66 | 0.97 | 0.026 | 0.68 | 0.57 | 0.80 | <0.001 | 0.58 | 0.50 | 0.66 | <0.001 |
| 2021 | 3.29 | 2.79 | 3.89 | <0.001 | 0.40 | 0.33 | 0.48 | <0.001 | 0.44 | 0.39 | 0.51 | <0.001 |

|  | **SS_freq** | | | | **FS_freq** | | | | **FF_freq** | | | |
| --- | --- | --- | --- | --- | --- | --- | --- | --- | --- | --- | --- | --- |
| source | odds ratio | 95% lower C.I. | 95%  upper C.I. | P-value | odds ratio | 95% lower C.I. | 95%  upper C.I. | P-value | odds ratio | 95% lower C.I. | 95%  upper C.I. | P-value |
| intercept | 0.38 | 0.33 | 0.44 | <0.001 | 0.43 | 0.38 | 0.50 | <0.001 | 0.03 | 0.02 | 0.04 | <0.001 |
| *District* |  |  |  |  |  |  |  |  |  |  |  |  |
| Darjeeling | 0.06 | 0.04 | 0.09 | <0.001 | 0.16 | 0.13 | 0.20 | <0.001 | 0.07 | 0.03 | 0.13 | <0.001 |
| East Champaran | 0.53 | 0.46 | 0.62 | <0.001 | 0.06 | 0.05 | 0.08 | <0.001 | 0.30 | 0.23 | 0.39 | <0.001 |
| Godda | 0.23 | 0.19 | 0.27 | <0.001 | 0.40 | 0.35 | 0.45 | <0.001 | 0.23 | 0.18 | 0.31 | <0.001 |
| Gopalganj | 2.34 | 2.01 | 2.71 | <0.001 | 0.40 | 0.34 | 0.46 | <0.001 | 0.57 | 0.43 | 0.75 | <0.001 |
| Katihar | 0.19 | 0.15 | 0.24 | <0.001 | 0.24 | 0.20 | 0.28 | <0.001 | 0.32 | 0.22 | 0.45 | <0.001 |
| Muzaffarpur | 1.65 | 1.43 | 1.89 | <0.001 | 0.52 | 0.46 | 0.59 | <0.001 | 0.22 | 0.16 | 0.31 | <0.001 |
| Purnia | 0.29 | 0.24 | 0.36 | <0.001 | 0.61 | 0.52 | 0.71 | <0.001 | 0.19 | 0.12 | 0.29 | <0.001 |
| Samastipur (reference) | |  |  |  |  |  |  |  |  |  |  |  |
| *Year* |  |  |  |  |  |  |  |  |  |  |  |  |
| 2017 (reference) |  |  |  |  |  |  |  |  |  |  |  |  |
| 2018 | 0.90 | 0.80 | 1.01 | 0.071 | 2.47 | 2.18 | 2.79 | <0.001 | 3.91 | 2.71 | 5.62 | <0.001 |
| 2019 | 0.89 | 0.79 | 1.00 | 0.051 | 2.31 | 2.05 | 2.60 | <0.001 | 4.59 | 3.22 | 6.56 | <0.001 |
| 2020 | 1.11 | 0.96 | 1.28 | 0.173 | 2.34 | 2.02 | 2.71 | <0.001 | 2.12 | 1.35 | 3.34 | 0.001 |
| 2021 | 0.85 | 0.73 | 0.98 | 0.029 | 1.80 | 1.56 | 2.09 | <0.001 | 3.65 | 2.38 | 5.58 | <0.001 |
