# Supplementary figures and images for: Molecular surveillance of insecticide resistance in Phlebotomus argentipes targeted by indoor residual spraying for visceral leishmaniasis elimination in India
Source: PLoS Negl Trop Dis. 2023 Nov 8;17(11):e0011734. doi: 10.1371/journal.pntd.0011734 (PMC10659200; doi:10.1371/journal.pntd.0011734)

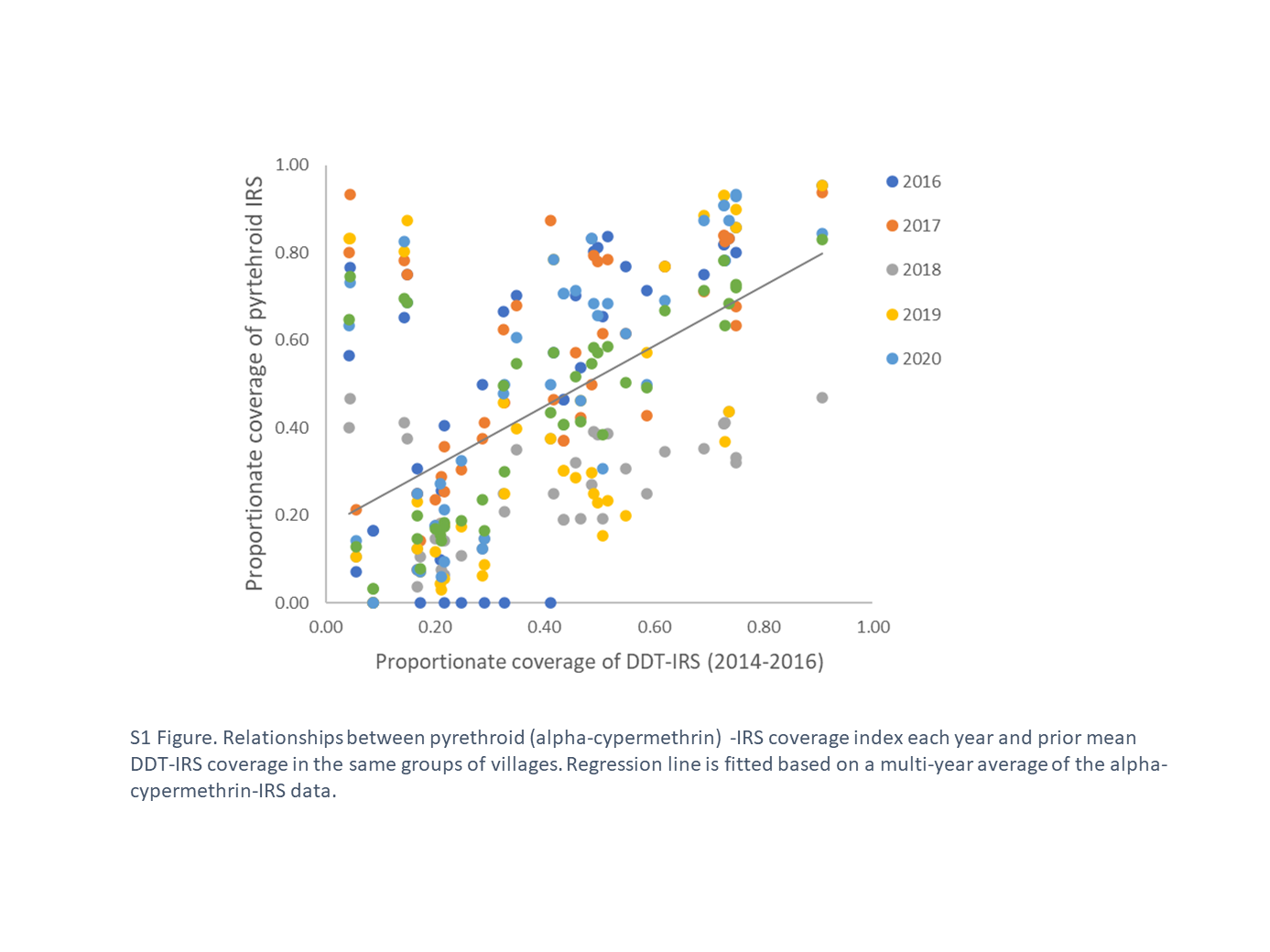

Supplement: S1 Fig — A linear regression line is fitted based on a multi-year average of the alpha-cypermethrin-IRS data. (TIF) [file pntd.0011734.s004.tif]
