# Supplementary material for: Molecular surveillance of insecticide resistance in Phlebotomus argentipes targeted by indoor residual spraying for visceral leishmaniasis elimination in India
Source: PLoS Negl Trop Dis. 2023 Nov 8;17(11):e0011734. doi: 10.1371/journal.pntd.0011734 (PMC10659200; doi:10.1371/journal.pntd.0011734)
Supplement: Attachment — Submitted filename: Responses to reviewers.docx [file pntd.0011734.s006.docx]

**Responses to reviewers**

To whom it may concern,

We thank the reviewers for their comments and suggested improvements to our manuscript. We have preserved the reviewer comments below and our responses are shown in red font. We have also made a few minor corrections where typographical errors not highlighted by the reviewers were detected, which are shown in the track changes version of the manuscript.

Thank you.

Mike Coleman.

Reviewer #2: The results section is well structured and provide enough details to understand the findings. The english is correct.
The qulaity of the figure can however be improved

Reviewer #3: The authors should consider making improvements to the quality of the figures in order to enhance clarity in the study. They should consider converting several tables, such as Table 1 and 2, into graphical representations (graphs) to better present the data. This approach can make the information more visually appealing and easier to interpret for the readers.

As suggested by both reviewers we have improved the quality and geographical scope of the map in Figure 1. We think the issue with perceived clarity of the other figures may stem from tiff conversion into the unified pdf format but if editorial staff perceive the quality to be insufficient for publication we can try to improve this. Tables 1 and 2 are GLM (model) results, which typically are displayed in tabular format and it is not clear how they could be converted into figures.

Reviewer #2: The conclusions should be amended to adress referee's comments as described below.

Reviewer #3: The data presented in the study supports the conclusions drawn by the authors, indicating that further progress towards pyrethroid resistance did not occur during the monitoring period for visceral leishmaniasis elimination. The limitations of the analysis are not explicitly described, but the authors suggest the need for additional research on genotype-phenotype associations and other resistance mechanisms. They discuss the value of molecular surveillance for the VL program and emphasize the importance of incorporating alternative insecticides into an integrated resistance management strategy. The authors' conclusions provide practical implications for controlling insecticide resistance and advancing the VL elimination program.

We thank the reviewer for these comments and have added a paragraph at the end of the discussion to clarify the major limitations of the analysis (see below)

--------------------


Reviewer #1: This is a clearly analysed and reported piece of work on an impressive amount of collected data. The methodology appears to be sound and my only comments are on minor typographical errors (listed below).

Line 85 - missing closing bracket and full stop. fixed

Line 126-127 - there appears to be 9 rather than 8 sites listed. Is “East” in the wring place? fixed

Line 130-131 - sentence needs altering (perhaps changing the comma to an ‘and’?) fixed

Line 153 - an extra closing bracket needs removing. fixed

Lines 174, 179 (and throughout) - perhaps use ‘GzLM’, to distinguish generalized from general linear model. Done

Line 414 - remove final “in”. Done

Figure 1 - would benefit from being inset in a larger map to indicate broader geographical location, and perhaps different shading of the districts studied. Done and attached as separate file.

Figure 4 - double-check East Champaran data (appears to show no change between 2017 and 2021). The data for the graph are correct and as we state in the manuscript (where the figure is cited) the figure shows little change in proportions between the start and end points of the collections.


Reviewer #2: Dear Editor,

The authors investigated the wide-scale spatio-temporal molecular variation of kdr mutations in Phlebotomus argentipes sampled from sentinel sites of north-eastern India during a period of intensive alpha-cypermethrin spraying for vector control. The idea was to assess whether changes in resistance marker frequency have occurred after the implementation of IRS with a pyrethoid. Overall, the authors found an increase in kdr genotype frequency between 2017 and 2018, but no significant changes in kdr composition afterward, with no evidence linking IRS activities to selection of any kdr genotypes (SS or FF). Despite the fact that the authors didn’t investigate the phenotypic resistance and the presence of resistance markers other than the Kdr, the paper provides very relevant information to guide decision making for vector control and resistance management.

Major Comment. The survey did not provide any information about the phenotypic resistance to alphacypermethrin in the sandflies collected during the period of treatment. Although I understand the complexity and difficulty to conduct bioassays according to WHO or CDC protocols, the authors don’t really know whether the overall level of resistance to alphacypermethrin (AC) has (or not) increased during the 4 years? Actually, the kdr was used as “a proxy” but other resistance mechanisms than kdr may be present and being under selection due to AC. This lack of information may be critical for the choice of insecticide to use for IRS in the coming years. Since the study focus on molecular markers only it would have been relevant to better explore the genome of the sandflies collected to assess potential regions/genes under selection using NGS and bioinformatic tools. This is particularly relevant since the kdr data did not provide outstanding results. The authors should also acknowledge this in the conclusion because it may be misleading to think that an absence of significant change in kdr may be sufficient to prevent alpha cypermethrin resistance to spread following successive rounds of IRS with this insecticide.

We thank the reviewer for this important comment and have added a paragraph at the end of the study to address these limitations. The limitation was already acknowledged in the last sentence of the conclusion.
